# Modelling clustering of vertically aligned carbon nanotube arrays

**DOI:** 10.1098/rsfs.2015.0026

**Published:** 2015-08-06

**Authors:** Clemens F. Schaber, Alexander E. Filippov, Thorsten Heinlein, Jörg J. Schneider, Stanislav N. Gorb

**Affiliations:** 1 Functional Morphology and Biomechanics, Zoological Institute, Kiel University, Am Botanischen Garten 1–9, 24118 Kiel, Germany; 2 Department of Electronic and Kinetic Properties of Non-linear Systems, Donetsk Institute for Physics and Engineering, National Academy of Sciences, 83114 Donetsk, Ukraine; 3 FG Systemdynamik und Reibungsphysik, Technische Universität Berlin, Institut für Mechanik, Sekr. C8–4, Raum M 122, Straße des 17. Juni 135, 10623 Berlin, Germany; 4 Technische Universität Darmstadt, Fachbereich Chemie, Eduard-Zintl-Institut für Anorganische und Physikalische Chemie, Alarich-Weiss-Straße 12, 64287 Darmstadt, Germany

**Keywords:** biomimetics, gecko-inspired attachment, friction, carbon nanotubes, clustering, vertically arranged carbon nanotube arrays

## Abstract

Previous research demonstrated that arrays of vertically aligned carbon nanotubes (VACNTs) exhibit strong frictional properties. Experiments indicated a strong decrease of the friction coefficient from the first to the second sliding cycle in repetitive measurements on the same VACNT spot, but stable values in consecutive cycles. VACNTs form clusters under shear applied during friction tests, and self-organization stabilizes the mechanical properties of the arrays. With increasing load in the range between 300 µN and 4 mN applied normally to the array surface during friction tests the size of the clusters increases, while the coefficient of friction decreases. To better understand the experimentally obtained results, we formulated and numerically studied a minimalistic model, which reproduces the main features of the system with a minimum of adjustable parameters. We calculate the van der Waals forces between the spherical friction probe and bunches of the arrays using the well-known Morse potential function to predict the number of clusters, their size, instantaneous and mean friction forces and the behaviour of the VACNTs during consecutive sliding cycles and at different normal loads. The data obtained by the model calculations coincide very well with the experimental data and can help in adapting VACNT arrays for biomimetic applications.

## 1. Introduction

Clustering of fibres of biomimetic attachment systems is a crucial factor limiting their effective adhesive forces [1,2]. Clustering occurs when the adhesive forces between the fibre tips are stronger than the forces required to bend the fibres [3–5]. In biological model organisms, different strategies have evolved to avoid clustering of the attachment hairs. In the gecko, one such strategy is the sophisticated hierarchic three-dimensional arrangement of the contact elements of the single foot hairs [6]. In insects, another strategy is the gradient in the material properties of the attachment hairs from stiff at the bottom to soft at the tip [7]. However, clustering of fine fibres might represent a way of stabilization of fibre arrays. A certain degree of clustering of the fibres of attachment devices may contribute to their adaptation to different macro and micro roughness substrates as previously suggested for the sub-digital anti-slip setae of chameleon feet [8].

**Figure 1.**
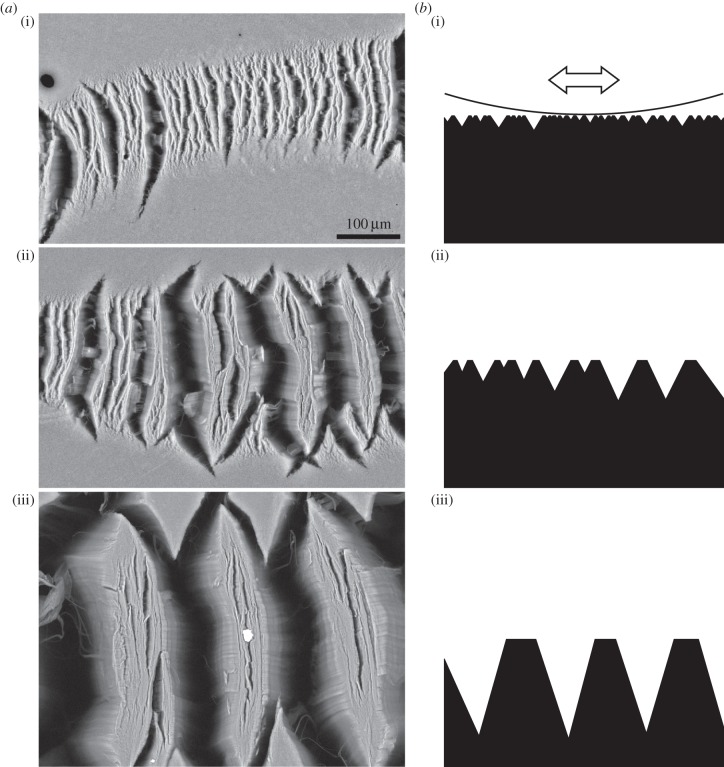
Clusters of VACNTs after friction tests with normal loads of (i) 289 µN, (ii) 673 µN and (iii) 3860 µN; top view scanning electron microscopy images (*a*) and schematic side views (*b*) of the surface of the arrays. The curve in *b*(i) depicts the surface of the spherical probe and the arrow points in sliding direction during friction tests.

Here we examined the clustering behaviour of 1 mm long arrays of vertically aligned multi-walled carbon nanotubes (VACNT) firmly bound to a substrate, which are good candidates for mimicking gecko foot hairs [9]. The thickness of single carbon nanotubes (CNTs) of between 5 and 20 nm is well in the range of that known for contact elements of gecko hairs [10]. The experimentally determined coefficient of friction on pristine surfaces of VACNT arrays is very high. It decreases with consecutive sliding cycles on the same location to still remarkably stable values. This decrease of friction is accompanied by clustering of the VACNTs, which stabilizes the mechanical properties of the arrays [11]. The size of the clusters depends on the normal force applied on the sample during friction tests (figure 1).

Previous simulations dealing with friction on vertically aligned fibrous materials mainly relied on finite-element models (FEMs) and on molecular dynamics approaches. One such model analysed static friction on a vertically aligned micro fibril array made of polydimethylsiloxane covered with a flat 4 µm film. The model predicted strong enhancement of friction compared with a control without the underlying fibrils [12]. Using molecular dynamics and FEM simulations, Hu *et al.* [13] showed an increase of shear force of VACNT blocks with increasing length of entangled tips of the single CNTs within the array. Lou *et al.* [14] simulated the interface between a probe (atomic force microscopy (AFM) tip, diameter 4 nm) and very short (1.5 nm) VACNTs arranged in a superlattice. They revealed that the stick–slip behaviour during friction tests is largely dominated by the penetration of the tip into the valleys between the single CNTs, which leads to strong interaction of the sides of the tubes with the sides of the probe. Using another atomic scale model, Landolsi *et al.* [15] calculated the stick–slip behaviour of a microspherical AFM probe on VACNT arrays with 30 nm protruding length of the CNTs.

To predict the results gained in our experiments, and to expand the knowledge about the experimentally not accessible interface between the probe and the surface of the VACNT arrays, we present a numerical model using the well-known Morse potential function [16]. The model calculates the van der Waals (VdW) interactions between the spherical probe and the bundles of VACNTs of the arrays to simulate their clustering and its effect on the coefficient of friction during repeated friction tests.

## 2. Material and methods

Experimental data were attained, as described before, by friction tests comprising five consecutive sliding cycles on the same location of the pristine surface of VACNT samples using a sapphire sphere as the probe (diameter 1.5 mm) [11]. Scanning electron micrographs of uncoated samples were taken using Hitachi TM3000 and S-4800 scanning electron microscopes at acceleration voltages of 3 kV.

The numerical simulation was carried out in Matlab R2012b (The MathWorks, Inc., Natick, MA, USA).

## 3. Theory

### 3.1. Simplification of the carbon nanotube array

**Figure 2.**
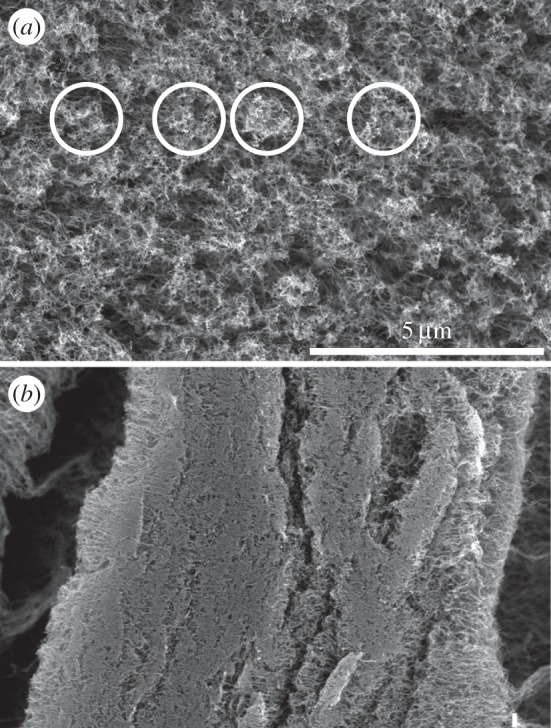
(*a*) Pristine surface of a VACNT array. Exemplary, some bunches of CNTs are surrounded by circles. (*b*) Clustered surface of the same sample after the friction experiment (same magnification as in (*a*)). Note that all bunches are condensed to one large single cluster.

In the pristine condition, the single nanotubes are supposed to be bundled together more or less uniformly to parallel bunches, each of which contains quite a large number of tubes fixed together (figure 2*a*). The base end of every tube, and of a bunch, is stationary and fixed to the substrate. The only degree of freedom remaining for a bunch is to rotate with respect to the fixation point. When an external force deflects the bunch, it tends to return to its original vertical orientation.

To avoid time-consuming calculations but still to simulate realistic collective behaviour of CNT arrays, we limit the model to the one-dimensional chain of bunches in the line of interaction with the external force. Each bunch is represented by its central point on top of the surface. For briefness, these points are further on called ‘bunches’. In the model, an effective elastic force, returning the bunches to their equilibrium vertical position, represents their rotation under load.

### 3.2. Formulation of the numerical model

#### 3.2.1. Elastic forces

For simplicity, the problem is reduced to a one-dimensional model where the deformation of the cluster only depends on its position on the direction of motion of the spherical indenter labelled by the coordinate *x*. Let us denote the alignment (an array in the numerical model) of the equilibrium positions of the tubes by 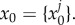 In the basic variant of the model, one can take all bunches as equivalent and placed equidistantly. In this case, 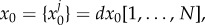 where *N* is the total number of bunches, *dx*_0_ = *L*/*N*, and *L* is the length of the system. As assigned above, when the instant positions of the tubes *x* = {*x^j^*} are shifted from their original positions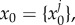 a set of elastic forces appear:3.1
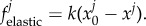


The phenomenological elastic constant *k* effectively simulates the rotational force appearing at the point where the rigid bunch attaches to the solid substrate. It is important to note once more that we reduced the description to a one-dimensional model. So all the values in the equation are scalar, and indices describe only the numbers in the array and not vector coordinates.

#### 3.2.2. Random distribution of different multi-walled nanotubes

For the real experimental set-up, the supposition of a regular lattice of equivalent bunches is not correct. Direct observation shows that there are quite a number of differently multi-walled CNTs with different diameters, and different distances between their centres (figure 2*a*). They form bunches in different proportions. For implementation of this feature into the model, the distance 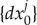 between nearest neighbours is randomly varied from one pair of the tubes to the other 
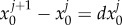
 with the total length of the system equal to the sum 
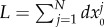
. The numerical realization of the array 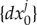 must contain all possible kinds of the distances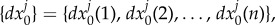 where *n* is the total number of possible distances randomly placed with statistical weights *P_k_* with *k* = 1,2, … , *n* corresponding to the empirical probability to find this realization in the real array 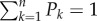. Therefore, our model accounts for the different size and elasticity of the tubes. Direct numerical simulation showed that this modification changes some particular quantitative results only, but does not influence the general qualitative behaviour. Taking this into account, to avoid overloading the text, the further description neglects these differences and is limited to the regular system.

#### 3.2.3. Interaction between the spherical probe and the carbon nanotube arrays

The single CNTs interact with the moving spherical probe (ball) and with each other by VdW forces. In turn, the spherical probe is moved by another elastic force produced by the external cantilever and interacts with the integral collective force from the bunches. These mesoscopic interaction forces must preserve the general properties of the Lennard-Jones potential of original intermolecular forces, but in the present numerical model, it is more convenient and in some sense even more realistic to simulate them as simply as possible using some effective potential.

One of the common representations for intermolecular potentials well suited for numerical simulations is the Morse potential [16]. In standard notations, it can be written as follows:3.2
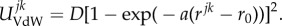


Here *D* is the depth of the potential minimum defined relative to the infinite intermolecular distance *r*^*jk*^ = |*x*^*j*^ − *x*^*k*^|, *r*_0_ is the position of the potential minimum and the parameter *a* = (*f*_VdW_/2*D*)^1/2^ controls the width of the potential at a given value of the force constant *f*_VdW_. It is defined as a derivation 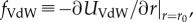 which is calculated at the potential minimum. Let us denote that ‘relative to the infinite intermolecular distance *r*^*jk*^ = |*x*^*j*^ − *x*^*k*^|’ written above designates one possible standard physical definition of potential energy formally calculated from its limiting value at distance going to infinity, and it does not mean actual infinite distance.

Interaction of bunches with the moving sphere is comparable with their mutual interaction. The corresponding potential can be written in the analogous form3.3
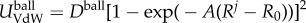
with another set of parameters *A*, *D*^ball^, *R*_0_ and the distances *R*^*j*^ = |*X* − *x*^*j*^| between the bunches and the instant positions of the rigid sphere with the coordinate of the centre of its mass *X*. All other forces, which remain out of our control, are included by an interaction of the system with an external ‘thermostat’, simulated by a set of *δ* correlated in time and space random sources: 〈*ζ*^*j*^〉 = 0, with intensity *σ*: 〈*ζ*^*j*^*ζ*^*k*^〉 = *σδ*_*jk*_, and energy dissipation described by the phenomenological damping constant *γ*.

The equations of motion can now be written in the final form:3.4
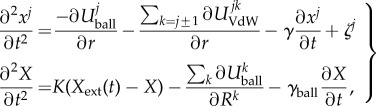
where *K* is the elastic constant and *X*_ext_(*t*) the instant position of the external cantilever.

For illustration, a movie captured during numerically solving this system of equations using Matlab software can be found in the electronic supplementary material.

## 4. Results

### 4.1. Qualitative behaviour of the system

We can forecast the following qualitative behaviour of the system before numerical simulation. In one limit, when the bunches are strongly attached, which results in almost no rotation from their original positions, and weak interaction between the bunches as well as with the sphere, we expect them to remain close to their initial positions 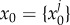.

In the opposite limit, strong interaction with the sphere will essentially perturb the positions especially of those bunches actually in close vicinity to the sphere. If the mutual interaction of the bunches in relation to the elastic force 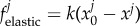 is strong enough as well, being once disturbed, the bunches will tend to a new equilibrium distance *r*_0_, which is normally considerably smaller than the initial *dx*_0_ = *L*/*N* in the array 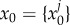. Let us note that the total number of the tubes is fixed, and the distance between them averaged over the whole system remains equal to *dx*_0_. The only compromise expected in this case is that the system must split into a number of relatively large clusters separated by depopulated gaps.

### 4.2. Functional concept of the model

The moving sphere pushes and attracts bunches dependent on their position in front or behind the sphere and gradually bundles them into clusters. Assembled bunches follow the sphere for a while and new bunches add on its way. When the size of the cluster surrounding the sphere becomes too large for a balance between the VdW and the elastic forces, the sphere cannot move the bunches altogether anymore. Some of them leave the sphere, and return as close as possible to their original positions.

**Figure 3.**
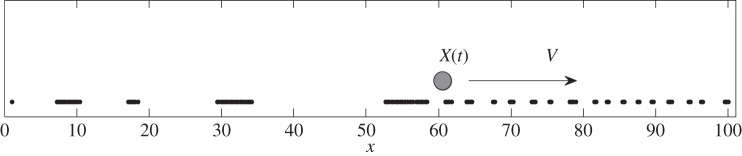
Conceptual snapshot of the process of clustering at an intermediate stage of the simulation. The tops of the bunches are shown by the black dots. The sphere is represented in its instant position *X*(*t*) by the grey circle. *V* is the instant velocity of the (external) cantilever scanning the system in both directions with positive and negative velocities *V*(*t*) = ±*V*.

Besides the interaction with the sphere, mutual interaction of nearest neighbours can produce spontaneous assembling of small groups containing two to three bunches in the present model. Our observation shows that this kind of instability of the perfect trial lattice appears for the soft system with an elasticity of *k* ≅ 0.01. Such little values of elasticity are necessary to model the experimentally observed strong effect of clustering (figure 2*b*). Figure 3 reproduces an intermediate view of such a system with partially formed groups of bunches in front of the sphere and extended clusters behind it.

### 4.3. Time-dependent scenario

**Figure 4.**
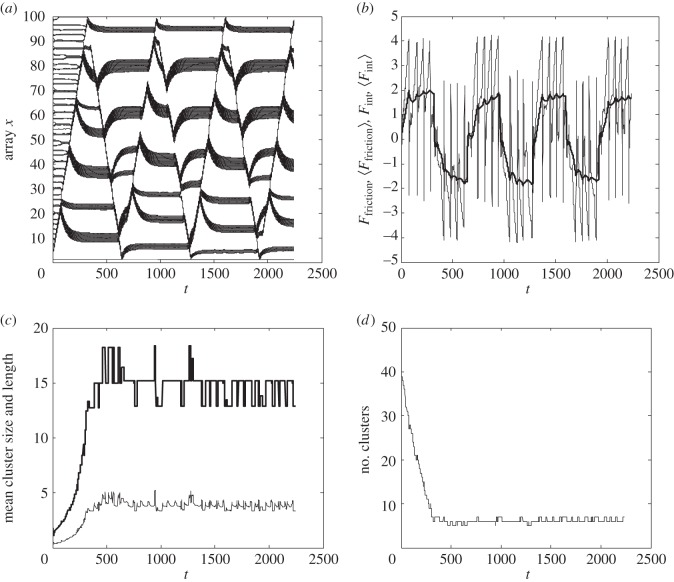
Typical evolution of the time-depending values in presence of clustering during five friction cycles. (*a*) Transformation of the array *x^j^* = *x^j^*(*t*). (*b*) Friction force (the force value averaged over each current scan is shown by the bold line). (*c*) Mean size (bold line) and length of the cluster. (*d*) Number of clusters.

The time-dependent scenario of the clustering can be recorded and quantitatively described by accumulation and plotting the complete set of time-depending arrays *x*^*j*^ = *x*^*j*^(*t*). Figure 4*a* shows a typical result of this procedure. One can see directly how the initially equidistant trajectories *x^j^*(*t*) evolve to the dense bundles corresponding to the clusters. Figure 4*b* shows the time-dependent friction force.

The ends of the clusters can formally be defined as those points where the instant distances *dx*_*jj*±1_(*t*) between the bunches are much longer than *dx*_0_ from one side and tend to *r*_0_ < *dx*_0_ on the other side of the given bunch *j*, for example *dx*_*jj*+1_*t* ≫ *dx*_0_ and *dx*_*jj*−1_ ≅ *r*_0_ < *dx*_0_ for the right end of the cluster.

The model allows constructively defining a procedure to calculate the total number of the clusters, the mean number of bunches per cluster (‘mean size of the cluster’) and the mean length, corresponding to the averaged distance between the left and right ends of the clusters. These time-depending values together with the corresponding number of clusters are presented in figure 4*c*,*d*, respectively. The relation between the ‘size’ and the length of the cluster gives a density of the tubes inside it, which is directly related to two spatial scales of the problem *dx*_0_ and *r*_0_, as well as to the mutual relation between elastic and interaction forces. Stronger rigidity of the original shifts the equilibrium distance between the bunches from *r*_0_ to the trial value *dx*_0_, and vice versa. Nanotubes freely rotating in their basal points favour the formation of larger clusters. This observation gives us good criteria to validate the simulation results.

**Figure 5.**
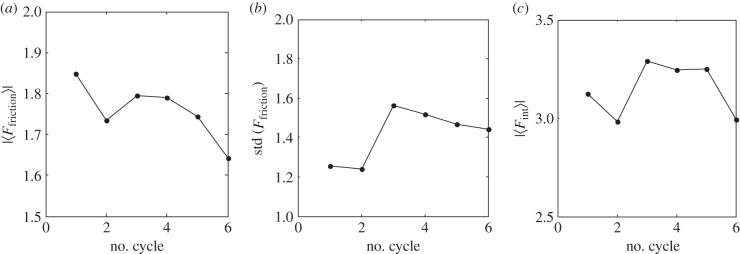
(*a*) Absolute friction force, (*b*) its standard deviation and (*c*) interaction strength between the sphere and the bunches (characterizing an effective mean contact area) integrated over each cycle.

Important information is also contained in the evolution of the time-depending friction force during each scan of the same region, as well as its standard deviation describing how pronounced the stick-and-slip effect is, and the variation of the size of the effective contact area. It characterizes a number of bunches close enough to the sphere to interact strongly with it, estimated on a base of absolute value of corresponding interaction force. From a physical point of view, it is interesting to accumulate the mean values of all of these variables between different consecutive scans and compare them. This information is in good correlation with experimental observation and summarized in figure 5.

### 4.4. Fitting of the model and the experimental data

**Figure 6.**
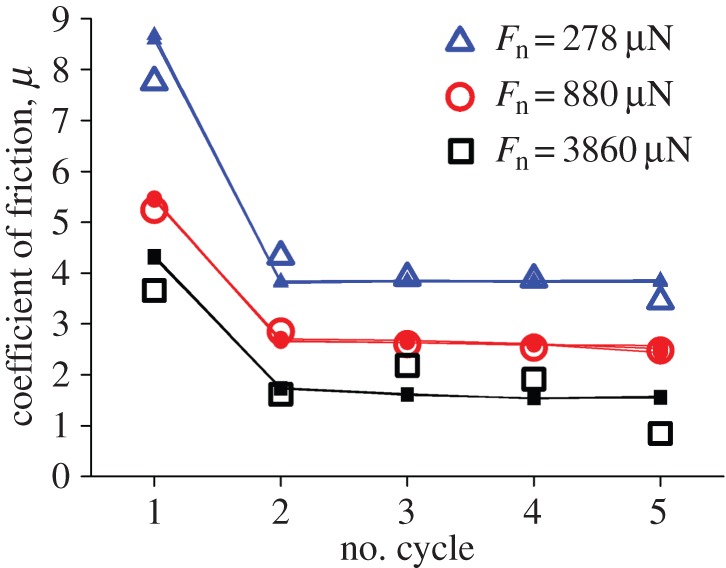
Modelled friction coefficients in five consecutive sliding cycles on the same array of bunches (small solid symbols) and experimentally determined values (open symbols) exemplary at three different mean normal loads *F*_n_. *ξ* was 0.61 to best match the experimental data for 278 µN normal load, 0.54 for 880 µN and 0.46 for 3860 µN. The model data overdrawn represent the results of three executions of the code with the same parameters. The differences between the runs are hardly visible.

To match the model with the empirical results, the factor *ξ* in the model adapts the strength of interaction between the ball and the bunches after the first sliding cycle at different experimental normal loads. The model well predicts the experimentally obtained friction coefficients and their decrease with consecutive sliding cycles (figure 6). *ξ* decreases monotonically with increasing mean normal load *F*_n_, from 0.61 at the smallest *F*_n_ of 278 µN down to 0.46 at the largest *F*_n_ of 3860 µN.

**Figure 7.**
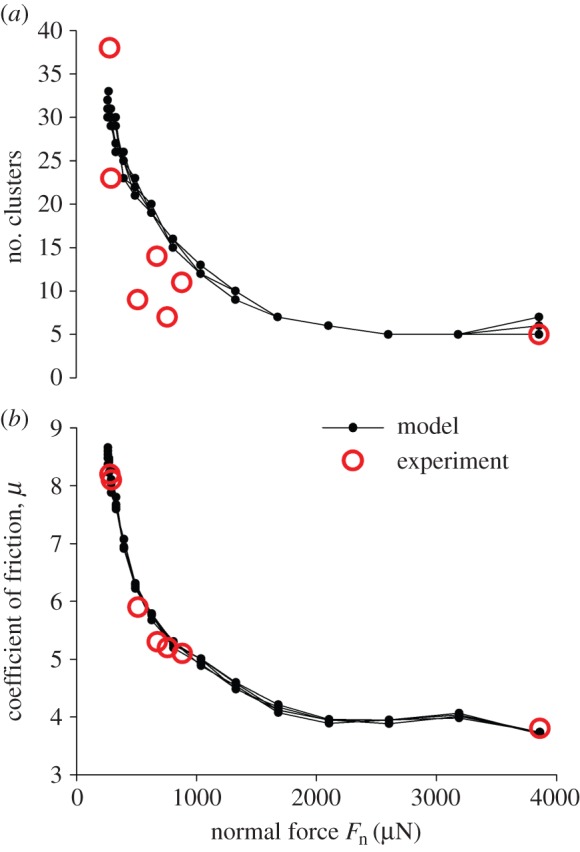
Predicted (small solid dots) and experimentally determined (open circles) values of (*a*) the number of clusters after five consecutive sliding cycles and (*b*) the friction coefficient at the first sliding cycle at different normal loads *F*_n_ on the surface of the arrays. The model data are overdrawn from six runs of the code with the same parameters.

Evaluating the model for the experimentally observed decrease of the number of clusters (corresponding to an increase of their sizes) with increasing normal loads, there is good agreement of the results (figure 7*a*). The same is true for the decrease of the friction coefficient with increasing normal force (figure 7*b*).

## 5. Conclusion

The agreement of simulated and experimental data allows for firm predictions beyond the existing experimental results. Applying the model, we can quickly calculate the coefficients of friction and the numbers of clusters of the VACNT arrays for any normal load required.

The model clearly shows that the clusters already form during the first sliding cycle of the friction tests, which goes along with the strong decrease of the friction coefficient from the first to the to the second cycle (figures 4 and 6). Therefore, there is strong evidence that the clusters structurally determine the stability of the high friction coefficient in consecutive cycles. The coefficient of friction practically does not change with normal loads exceeding approximately 2 mN in our friction testing set-up, as does the number of clusters along the given sliding length (figure 7).

Regarding possible applications of the VACNT arrays as biologically inspired anti-slip and attachment devices based on fibrillar adhesion, the simulation shows that the preconditioning leading to stable friction values is largely done after the first cycle. Furthermore, the model implies that VACNT arrays can handle a wide range of loads for applications where high friction is needed.

An interesting application could be the implementation into devices used for transfer printing of microelectronic structures as was proved in principle for real gecko foot hairs [17] and gecko-inspired elastomeric microflaps [18] as structures for switchable and reversible adhesion.
